# Biomolecular study of human thymidylate synthase conformer-selective inhibitors: New chemotherapeutic approach

**DOI:** 10.1371/journal.pone.0193810

**Published:** 2018-03-14

**Authors:** Hala O. El-Mesallamy, Hekmat M. El Magdoub, James M. Chapman, Nadia M. Hamdy, Mona F. Schaalan, Lamiaa N. Hammad, Sondra H. Berger

**Affiliations:** 1 Department of Biochemistry, Faculty of Pharmacy, Ain Sham University, Cairo, Egypt; 2 Department of Biochemistry, Faculty of Pharmacy, Misr International University, Cairo, Egypt; 3 Department of Pharmaceutical and Biomedical Sciences, College of Pharmacy, University of South Carolina, Columbia, SC, United States of America; Consejo Superior de Investigaciones Cientificas, SPAIN

## Abstract

Thymidylate synthase (TS) is a well-validated target for the therapy of adult cancers. Propane-1,3-diphosphonic acid (PDPA) has significant inhibitory properties against human thymidylate synthase (hTS) relative to mouse TS which is not predicted to adopt an inactive conformer. The current research aims to identify novel, lead inhibitors of hTS and examine the prediction that they bind selectively to hTS enzymes existing in different conformational equilibria. Conformer-selectivity was evaluated through performing activity inhibition studies, as well as intrinsic fluorescence (IF) studies in comparison to the known orthosteric inhibitor raltitrexed (RTX). Human TS was isolated from recombinant bacteria expressing either native hTS, capable of conformational switching, or an actively stabilized mutant (R163K-hTS). The examined test compounds were rationally or virtually predicted to have inhibitory activity against hTS. Among these compounds, glutarate, N-(4-carboxyphenyl) succinamic acid, and diglycolic anhydride showed higher selectivity towards native hTS as compared to R163K-hTS. The active site inhibitor RTX showed significantly higher inhibition of R163K-hTS relative to hTS. Targeting hTS via conformational selectivity represents a future approach for overcoming reported resistance towards active-state TS analogs.

## Introduction

Thymidylate synthase (TS) is a well-validated target for the therapy of adult cancers including gastrointestinal, breast, pancreatic, and head and neck cancers [1]. At elevated levels, TS exhibits oncogenic behavior [2]. In the TS-catalyzed reaction, thymidylate (dTMP) is formed from deoxyuridylate (dUMP) using N^5^, N^10^ methylene tetrahydrofolate (mTHF) as the methyl donor. Analogs of TS substrates are utilized clinically as cancer chemotherapy, including, 5-fluorouracil, capecitabine, pemetrexed, and raltitrexed (RTX) [3]. Upon binding to TS, inhibitory complexes are formed that are catalytically inactive, resulting in depletion of dTMP. Such a thymine-less state is lethal to most actively dividing cells, and thus TS is an ideal target for anticancer therapy. Paradoxically, exposure to TS inhibitors is associated with elevation in TS levels. The binding of the inhibitor to TS is associated with increased stability of the enzyme to degradation and increased TS protein synthesis due to translational de-repression [4,5]. Elevation in TS levels, after exposure to inhibitors, is postulated to contribute to the resistance that is reported in patients receiving TS-targeted chemotherapy [6]. High-resolution crystal structures provided evidence for the existence of native hTS in active and inactive conformations based on the position of loop 181–197 containing cysteine (Cys) at position 195, the nucleophile involved in catalysis [7, 8]. The binding of RTX to hTS resulted in complexes that crystallized in a closed, active conformation [9]. This led to the hypotheses that stabilization of an active conformation underlies the elevation of hTS after inhibition, and that compounds that stabilize an inactive conformation may provide a novel approach for inhibiting TS. Superpositioning of crystal structures of the two conformations led to identification of three residues that are predicted to stabilize or destabilize each state [7, 8]. Substitutions at these sites resulted in mutant TS enzymes that exhibited approximately 1–25% (inactive) and 148% (active) of the catalytic activity of native hTS, respectively [10]. Relative to the active-stabilized mutant, designated R163K-hTS, mutants stabilized in an inactive conformation, exhibited lower intrinsic fluorescence (IF), increased thermostability, and resistance to the orthosteric inhibitor RTX. The change in IF is attributed to presence of a tryptophan (Trp) residue at position 182 of hTS. Previous modeling showed that the position of the indole moiety of Trp 182 differs between the active and inactive conformations by about 5 Å, whereas the positions of other Trp residues were reported to be similar in both conformers [8, 11].

Inspection of the crystal structures of hTS showed that an inactive conformation of loop 181–197 is stabilized by three or four sulfate or phosphate ions [12]. The distances between these ions, 6.5 Å, 9.5 Å, and 9.9 Å, suggested that bifunctional acidic ligands may have stronger propensity to stabilize the inactive conformer through ionic bonds with basic amino acids. Diphosphonates with 3–6 carbon linkers, which have distances between phosphonate moieties in the desired range, were tested for inhibitory properties against hTS. One of the inhibitors, propane-1,3-diphosphonic acid (PDPA), exhibited higher inhibitory potency against hTS relative to mouse TS, which is not predicted to populate the inactive conformer observed in hTS [13]. One goal of our research is to identify novel, lead inhibitors of hTS that bind to hTS distinctly from active-state inhibitors such as RTX. The selected compounds are chemotypes of PDPA or are predicted to bind to an inactive conformer of hTS. Conformational selectivity was evaluated by analyzing their effects on the catalytic activity and IF of native hTS and an active-stabilized mutant, R163K-hTS. Several of the tested compounds exhibited higher potencies against native hTS than R163K-hTS, a pattern distinct from RTX. At concentrations that cause maximal inhibition of hTS, they were more effective in inducing shifts in IF than RTX. Our data indicate that these compounds are novel inhibitors of hTS that behave distinctly from current clinically used inhibitors.

## Materials and methods

### Bacterial strains, plasmids and enzyme purification

The *Escherichia coli* strain TX61 (thyA^-^) containing a kanamycin resistant gene and the pTS080 plasmid expressing hTS and containing tetracycline and ampicillin resistant genes, were generously provided by Walter S. Dallas (Glaxo Wellcome, Research Triangle Park, NC). TX61 was created by transposon-mediated mutagenesis and lacks endogenous TS activity [14]. Creation of a mutant hTS with substitution of amino acid at position 163 of hTS has been previously reported [7]. TX61 transformants were grown in LB broth, and both hTS and R163K-hTS in cell extracts, were purified by AKTA® FPLC system (Pharmacia, Piscataway, NJ, USA) using Blue-Sepharose and Q-Sepharose columns with degassed buffers containing Tris, KCl, and mercaptoethanol, as described previously [15]. Briefly, bacterial lysates were run in the ACTA® FPLC system, using Blue-Sepharose columns. Eluted proteins were detected by monitoring UV absorption at 280 nm. The TS fractions were detected by monitoring absorbance change at 340 nm using a kinetic spectrophotometric assay based on the rate of conversion of mTHF to DHF [15]. TS fractions were washed with buffer, desalted and concentrated. TS enzymes were then passed through Q-Sepharose columns, eluted, desalted and concentrated. Enzyme purity was assessed by loading 1 μg denatured enzyme on 12% sodium dodecylsulfate-polyacrylamide gels for electrophoresis [16], using protein standards of molecular weights 20–100 kDa (Bio-Rad, CA, USA). Each gel was run at 110 volts for approximately 80 min using a Mini-Sub® Cell GT System (Bio-Rad). Proteins were visualized using 0.25% w/v Coomassie brilliant blue staining [17] (S1 Fig). The concentrations of purified TS proteins were determined by absorption at 280 as described previously [18]. Purified proteins were stored in 50 mM Tris-HCl, pH 7.4, containing 0.2% mercaptoethanol, 1 mM EDTA, and 15% glycerol.

### Catalytic activity assays

The k*_cat’_s* of both hTS and R163K-hTS were calculated according to the change in absorbance at 340 nm, as a result of the conversion of mTHF to DHF [15]. The activities of purified hTS and R163K-hTS were 1.137 ± 0.026 and 1.467 ± 0.031 sec-^1^, respectively.

**Fig 1:**
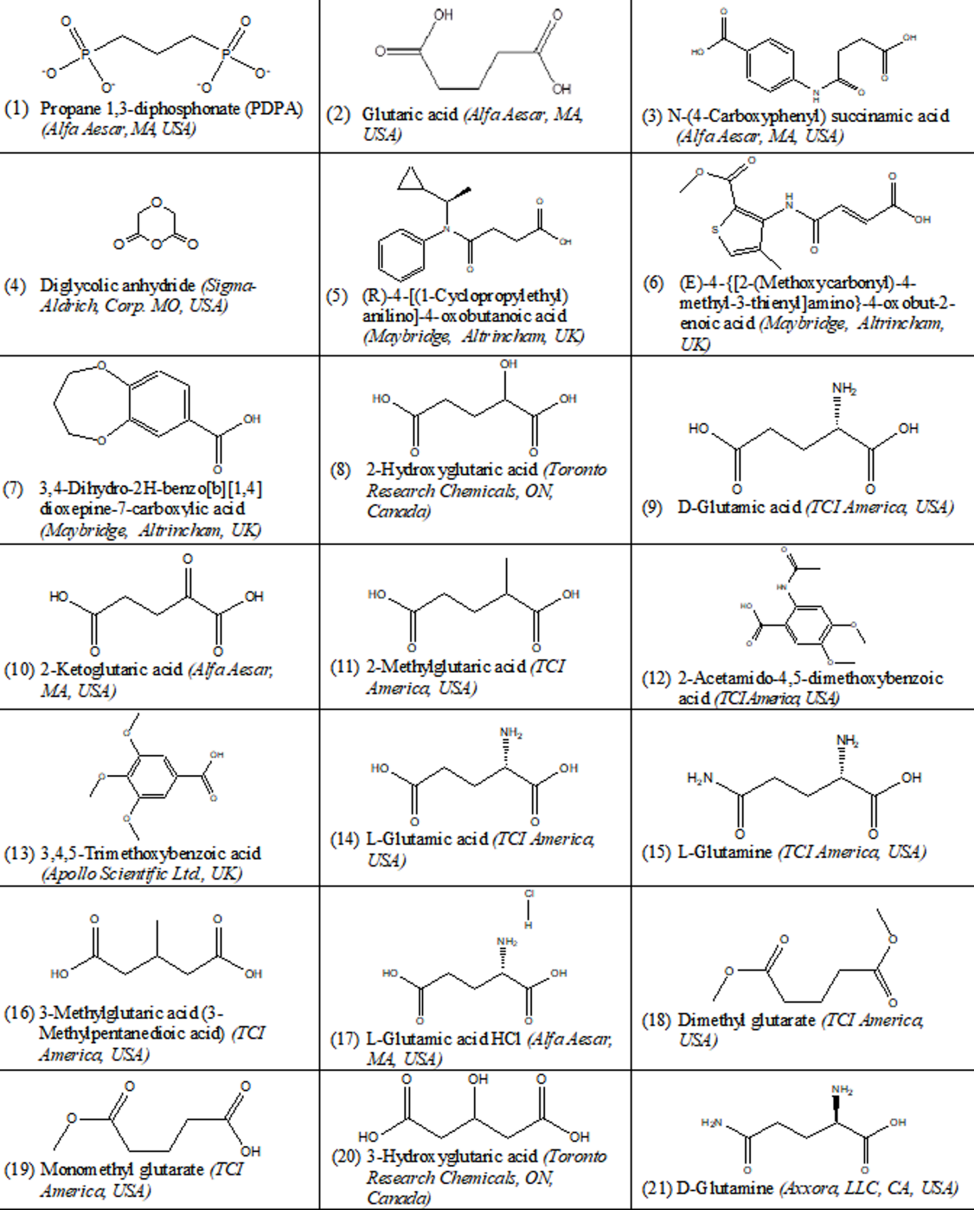
The examined test compounds for TS inhibition, their reference numbers, structures, and manufactures.

In the inhibition studies, catalytic activities of hTS and R163K-hTS were analyzed by a radioactivity assay, as described previously [19]. This assay measures the release of labeled tritium from 0.075 Ci/mmol 5-[^3^H] dUMP during conversion to dTMP, using 100 nM TS enzyme in reactions containing 50 mM Tris-HCl pH 7.4, 100 μM dUMP, 20 mM β-mercaptoethanol, 150 mM KCl, and 25 mM MgCl_2_. Reactions were initiated by the addition of 220 μM mTHF and terminated at the indicated time intervals with the addition of 1.3 N tricarboxylic acid in 5 mM dUMP. Unreacted radiolabel was removed by charcoal adsorption and centrifugation. The quantity of tritium released was detected by scintillation counting using a Tri-carb model 2900 TR (Packard Biosciences, Waltham, MA, USA). The procedure was repeated in presence and absence of different concentrations of 21 test compounds. Percent activity was calculated by dividing the slope of the line (representing activity versus time in the presence of different test compounds) by that of the control. Blanks were used to determine residual radioactivity in solution, and standards were used to determine total solution radioactivity in the absence of charcoal. For compounds solvated in DMSO, the final concentration of DMSO in reactions was 5%. In a second approach, we compared the effect of the same test compound at the same concentration on the activities of each enzyme. The significant inhibition of R163K-hTS relative to that of native hTS using the same concentration of the same test compound is denoted by @. Test compounds are shown in Fig 1. The majority of compounds are carboxyl isosteres of PDPA, some of which contain functional groups in the alkyl chain or have modified carboxyl groups. Heterocyclic compounds were derived from structure-based modeling [20]. Briefly, ligand structures were obtained from indicated manufacturers in *.sdf formats. The X-ray crystal structure 1 HW3 of a dimer of an inactive conformation of hTS was overlapped with that of PDPA-bound hTS monomer 2ONB obtained by soaking hTS crystals in an inactive conformation [13]. Docking was performed using Ligand Fit provided by Discovery Studio 2.1-Accelrys (San Diego, CA). From 13,750 compounds, 6 potential leads [(3),(5),(6),(7) (12),(13)] were selected as test compounds.

### Intrinsic fluorescence (IF) studies

One approach for monitoring hTS conformational changes upon ligand binding is by measuring IF. Test compounds were added to the enzyme in increments not exceeding 10% of the total volume, and fluorescence data were obtained by excitation at 295 nm and emission scanning from 310 to 470 nm at room temperature using Spectra Max M2e (Molecular Devices, Inc., Sunnyvale, CA, USA), and recorded in terms of relative fluorescence units (RFU), according to the method of Phan et al. [11]. Minimal fluorescence was observed in the buffer alone and studies were conducted over short periods of time to maintain enzyme integrity. Change in IF (ΔF) was calculated by subtracting the fluorescence units at 340 nm of the enzyme ± inhibitor, using the following equation;

ΔF340 nm = RFU340 nm (enzyme)—RFU340 nm (enzyme plus test compound).

### Statistical analysis

All data were submitted to a computerized statistical treatment using “GraphPad Prism” (for Windows, Version 5), and the Microsoft computer program for analysis. All values were expressed as means ± S.D. for quantitative measures. Statistical analysis was performed using one-way ANOVA followed by Dunnette post hoc test for comparing the means of normally distributed data to the controls. Meanwhile, Bonferroni post hoc test was used for comparing the means of normally distributed data of different groups to each other. Two-way ANOVA followed by Bonferroni post hoc test were used for comparing the means of the parametric data between and within groups showing significant differences. The association between normally distributed parameters was determined using the Pearson’s correlation coefficient. The probability of error (*P*-value) was considered statistically significant at *p* ≤ 0.05.

## Results

### Catalytic activity assay

The k_*cat*_ of R163K-hTS was 129% that of native hTS, as expected of an enzyme stabilized in an active state. Upon comparing the inhibitory potencies of PDPA and its selected chemotypes on hTS enzyme activity, it was found out that 1 mM of PDPA, glutaric acid, N-(4-carboxyphenyl) succinamic acid, diglycolic anhydride, (*R*)-4-((1-Cyclopropylethyl)(phenyl)amino)-4-oxobutanoic acid, (E)-4-((2-(methoxycarbonyl)-4-methythiophen-3-yl)amino)-4-oxobut-2-enoic acid, 3,4-dihydro-2H-benzo[b][1,4]dioxepine-7-carboxylic acid, (compounds (1),(2),(3),(4),(5),(6) and (7), respectively) resulted in a significant decrease in hTS activity as compared to the control. Upon using 3 mM of these compounds, it was evident that compounds (1), (3), and (4) were the most potent resulting in almost complete inhibition of hTS activity. Meanwhile, using 10 mM of any of these test compounds resulted in a complete inhibition of hTS, as shown in Table 1. Compounds (3), (5), (6) and (7) were among those compounds dissolved in DMSO. DMSO had no significant effect on hTS activity at the concentrations utilized in these studies.

**Table 1:** Effect of 1, 3, and 10 mM of different test compounds on hTS % activity.

Cpd. # / Conc.		1 mM	3 mM	10 mM
**Control**	**% Activity** **Mean ± SD**	100 ± 2.27		
**(1)**		80.6*± 5.00	0.53*^¥^ ± 0.75	0.93* ± 0.70
**(2)**		84.3*± 2.80	33.5*^¥^^‡^ ± 2.22	0.70* ± 1.54
**(3)**		76.8*± 4.75	0.20*^¥^ ± 0.36	0.17* ± 0.96
**(4)**		77.8*± 3.97	1.67*^¥^ ± 1.56	0.57* ± 0.74
**(5)**		84.7*± 2.80	69.7*^‡^ ± 1.25	0.17* ± 0.32
**(6)**		84.7*± 3.09	71.0*^‡^ ± 5.04	0.40* ± 0.46
**(7)**		83.5*± 3.25	73.3*^‡^ ± 1.32	0.57* ± 0.50

Values represent hTS activity in presence of different concentrations of selected test compounds as % activity of control. The control value represents hTS enzyme activity in the absence of any of the test compounds. The data are the means of 3 separate determinations of the slopes of the activity versus time.

* Significant difference from control, *at P ≤ 0*.*05*.

¥ *Significant difference from compounds (5)*, *(6) and (7) at the same concentration (3 mM)*, *at P ≤ 0*.*05*.

‡ *Significant difference from compounds (1)*, *(3) and (4) at the same concentration (3 mM)*, *at P ≤ 0*.*05*.

Upon comparing the inhibitory potencies of different concentrations of the compounds, that produced maximal inhibition of hTS (compounds (1), (2), (3) and (4)) on R163K-hTS, it is obvious that using 1 mM of compounds (1), (2) and (4) did not produce any significant inhibition of R163K-hTS. Using 3 mM of the four test compounds produced significant inhibition of R163K-hTS activity relative to its control. The decrease in % activity ranged from about 42.6% with compound (1) to 73% with compound (3). Compound (3) inhibitory potency on R163K-hTS activity was significantly higher than the other three compounds. When comparing the inhibitory potencies of these four compounds on R163K-hTS relative to hTS, it turned out that upon using 3 mM, the four test compounds were significantly less potent inhibitors of R163K-hTS relative to hTS. At the 10 mM concentration selectivity was lost and almost complete inhibition of both conformers was evident, as shown in Table 2. Meanwhile, the inhibitory potency of RTX on R163K-hTS was significantly higher than that observed with hTS at 0.3, 1 and 3 μM, by 26.3%, 22.5% and 13.4% respectively (Table 3).

**Table 2:** Effect of 1, 2, 3 and 10 mM of compounds (1), (2), (3) and (4) on hTS versus R163K-hTS % activity.

Cpd. # Conc.		1 mM	3 mM	10 mM
		% Activity Mean ± SD		
**Cpd. (1)**	**hTS**	80.4 ± 5.20	0.25 ± 0.43	0.66 ± 1.22
	**R163K-hTS**	99.9^@^ ± 1.80	57.4^@^ ± 3.90	0.39 ± 0.53
**Cpd. (2)**	**hTS**	84.2 ± 5.25	34.2 ± 2.98	0.30 ± 0.58
	**R163K-hTS**	94.3^@^ ±1.62	50.3^@^ ± 3.90	-0.54 ± 0.90
**Cpd. (3)**	**hTS**	76.9 ± 4.94	0.17 ± 0.86	0.96 ± 1.75
	**R163K-hTS**	76.0 ± 4.21	27.0^@^ ± 4.49	0.19 ± 0.86
**Cpd. (4)**	**hTS**	77.3 ±3.54	0.89 ± 0.42	0.26 ± 1.10
	**R163K-hTS**	100.5^@^± 2.15	47.1^@^ ± 6.38	-0.25 ± 1.15

Values represent enzyme activity as % of control in presence of different concentrations of selected test compounds. The data are the means of 3 separate determinations.

^@^ *Significant difference from hTS*, *at the same concentration*, *at p ≤ 0*.*05*.

**Table 3:** Effect of different concentrations of RTX on hTS versus R163K-hTS % activity.

RTX conc. / TS conformer		hTS	R163K-hTS
**Control**	**% Activity** **Mean ± SD**	100.0 ± 6.84	100.0 ± 5.84
**0.3 μM**		81.6* ± 2.40	55.3*^@^ ± 3.49
**1 μM**		57.7* ± 4.96	35.2*^@^ ± 4.21
**3 μM**		30.1* ± 3.46	16.7*^@^ ± 2.86
**10 μM**		5.9* ± 2.07	4.4* ± 1.15

Values represent enzyme activity as % of control in presence of different concentrations of RTX. Control values represent enzyme activities in the absence of any of the test compounds. Values are the means of 3 separate determinations.

*Significant difference from its control, *at p ≤ 0*.*05*.

^@^*Significant difference from hTS using the same concentration of RTX*, *at p ≤ 0*.*05*.

### Intrinsic fluorescence (IF) studies

The effects of different concentrations of compounds (1), (2), (3), and (4) on IF of hTS versus R163K-hTS are shown in Table 4. It is shown that the highest concentration used of compound (3) produced almost 95% decrease in IF of hTS, compared to approximately 53–60% decrease with compounds (1), (2) and (4).

**Table 4:** Effect of 1, 2, 3 and 10 mM of compounds (1), (2), (3) and (4) on % ΔF of hTS versus R163K-hTS.

Cpd. # Conc.		1 mM	2 mM	3 mM	10 mM
		%ΔF Mean± SD			
**Cpd. (1)**	**hTS**	12.2 ± 1.12	48.2^b^ ± 3.11	53.6^b^ ± 5.10	59.9 ± 2.14
	**R163K-hTS**	3.1^*@*^ ± 0.99	50.6^e^ ± 2.40	55.0^e^ ± 2.84	61.7^e^ ± 1.87
**Cpd. (2)**	**hTS**	7.4 ± 0.46	12.9 ± 1.12	37.1 ± 0.70	56.0 ± 5.92
	**R163K-hTS**	0.37 ± 0.29	0.53^*@*^ ± 0.40	26.8^*@*^ ± 2.09	53.1 ± 2.50
**Cpd. (3)**	**hTS**	21.2 ^a^^b^^c^ ± 2.19	43.0^b^ ± 2.58	73.4^a^^b^^c^ ± 0.95	95.2^a^^b^^c^ ± 1.54
	**R163K-hTS**	24.4^d^^e^^f^± 2.05	45.0^d^^e^ ± 2.25	77.2^d^^e^^f^± 1.82	90.8^d^^e^^f^± 3.22
**Cpd. (4)**	**hTS**	13.7 ± 2.65	45.7^b^ ± 1.87	49.6^b^ ± 2.90	52.8 ± 1.68
	**R163K-hTS**	3.5^*@*^ ± 0.23	46.8^e^ ± 2.35	47.5^d^^e^± 2.42	52.0^e^ ±1.76

Values represent % ΔF of hTS from its control in presence of different concentrations compounds (1), (2), (3) and (4). The data are the means of 3 separate determinations.

^*@*^ *Significant difference from hTS at the same concentration*, *at p ≤ 0*.*05*.

*a Significant difference from compound (1) on hTS at same concentration*, *at p ≤ 0*.*05*.

*b Significant difference from compound (2) on hTS at same concentration*, *at p ≤ 0*.*05*.

*c Significant difference from compound (4) on hTS at same concentration*, *at p ≤ 0*.*05*.

*d Significant difference from compound (1) on R163K-hTS at same concentration*, *at p ≤ 0*.*05*.

*e Significant difference from compound (2) on R163K-hTS at same concentration*, *at p ≤ 0*.*05*.

*f Significant difference from compound (4) on R163K-hTS at same concentration*, *at p ≤ 0*.*05*

The effect of different concentrations of compounds (1), (2), (3) and (4) on % ΔF of R163K-hTS is shown in Table 4. The data demonstrate that at both, the lowest (1 mM) and highest (10 mM) concentrations used, compound (3) was the most potent (24.4% and 90.8% change in IF, respectively).

**Fig 2:**
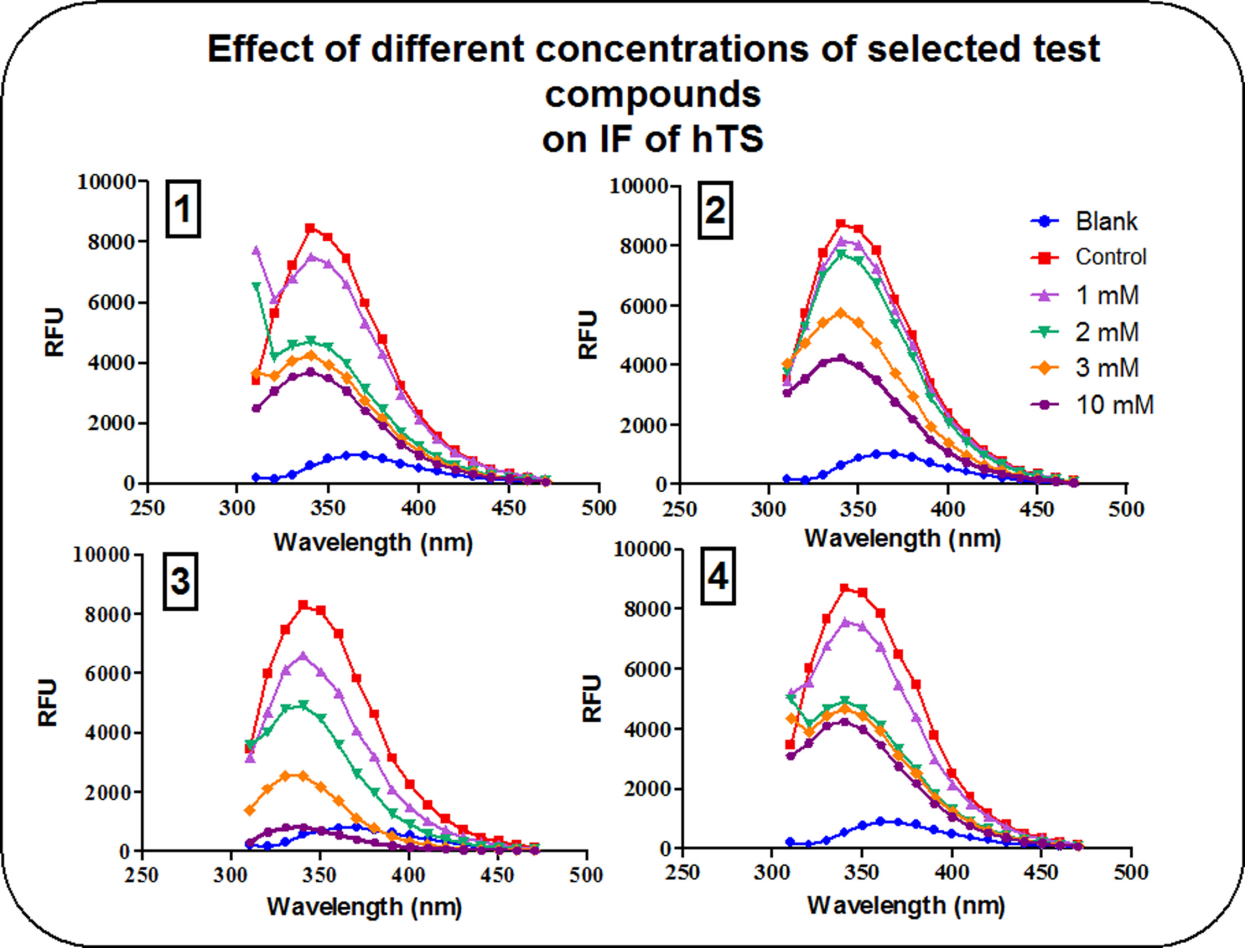
Effect of different concentrations of compounds (1), (2), (3) and (4) on IF of hTS. Controls contained the enzyme in buffer without any of the test compounds, while blanks were performed using buffer only.

**Fig 3:**
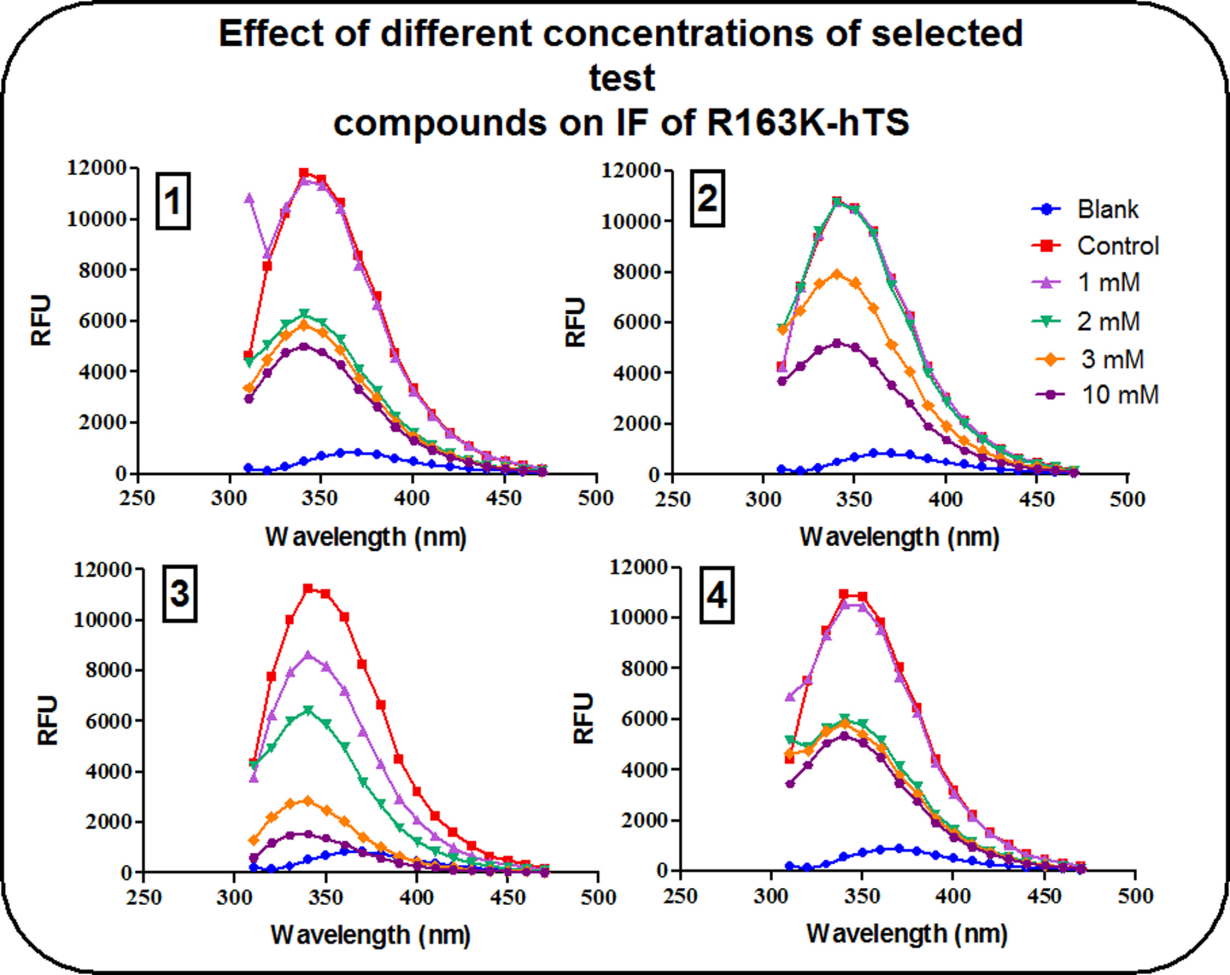
Effect of different concentrations of compounds (1), (2), (3) and (4) on IF of R163K-hTS. Controls contained the enzyme in buffer without any of the test compounds, while blanks were performed using buffer only.

At 1 mM, compounds (1), (2) and (4) were able to decrease IF intensity of hTS by about 7–14%. Meanwhile, their effect on R163K-hTS did not exceed 3.5%. The effect of compound (3) at a similar concentration was much more pronounced, as it was able to decrease IF intensity of hTS by about 21% compared to 24% with R163K-hTS, a difference that is not statistically significant. At the level of maximum inhibition of IF intensity, the change observed with compounds (1), (2) and (4) ranged from 52–62% for both hTS and R163K-hTS, while the effect of compound (3) ranged from 91–95% for both conformers as shown in Figs 2 and 3, respectively.

In order to examine whether the two enzyme conformers respond differently towards active site inhibitors, IF was measured in presence and absence of different concentrations of RTX. The data indicate that the intensity of IF was affected by the concentration of RTX used, while λmax remained unchanged at 340 nm as shown in Table 5.

**Table 5:** Effect of different concentrations of RTX on % ΔF of hTS versus R163K-hTS.

Conc. / TS conformer		hTS	R163K-hTS
**0.3 μM RTX**	**% ΔF** **Mean ± SD**	4.4 ± 0.53	6.1 ± 0.51
**1 μM RTX**		8.8 ± 0.64	15.4 ^@^ ± 1.92
**3 μM RTX**		18.9 ± 2.99	31.6 ^@^ ± 0.97
**10 μM RTX**		33.4 ± 5.72	57.4 ^@^ ± 1.66

Values represent % ΔF of hTS versus that of R163K-hTS in presence of different concentrations of RTX. The data are the means of 3 separate determinations.

^*@*^ *Significant difference from hTS at the same RTX concentration*, *at p ≤ 0*.*05*.

The effect of RTX on R163K-hTS was about 1.75, 1.67 and 1.72 fold that of hTS upon using 1, 3 and 10 μM RTX, respectively, as shown in Table 5. All of these values represented statistically significant differences.

**Fig 4:**
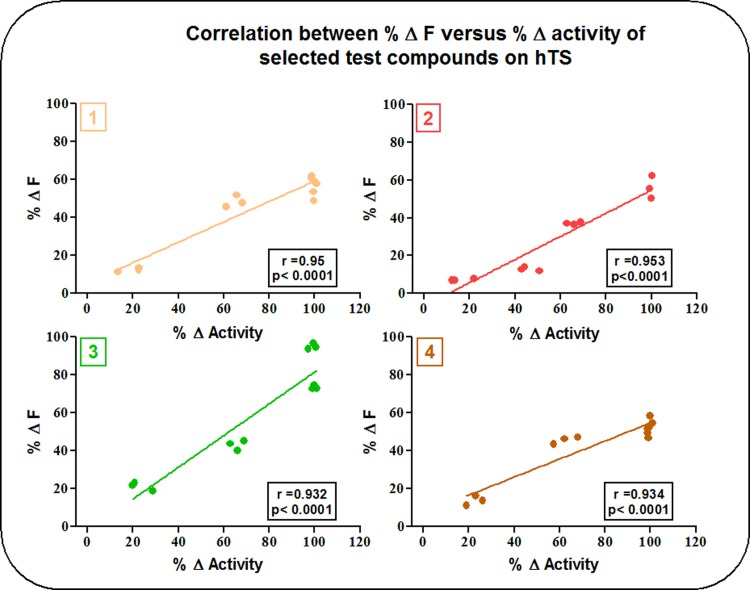
Correlation between activity inhibition and IF studies of compounds (1), (2), (3) and (4) on hTS.

The results of the current study showed that compounds (1), (2), (3) and (4) had a strong correlation between activity inhibition of hTS, and the change in its IF. Values of r ranged from 0.953 with compound (2) to 0.932 with compound (3) as demonstrated in Fig 4 with p-values < 0.0001.

**Fig 5:**
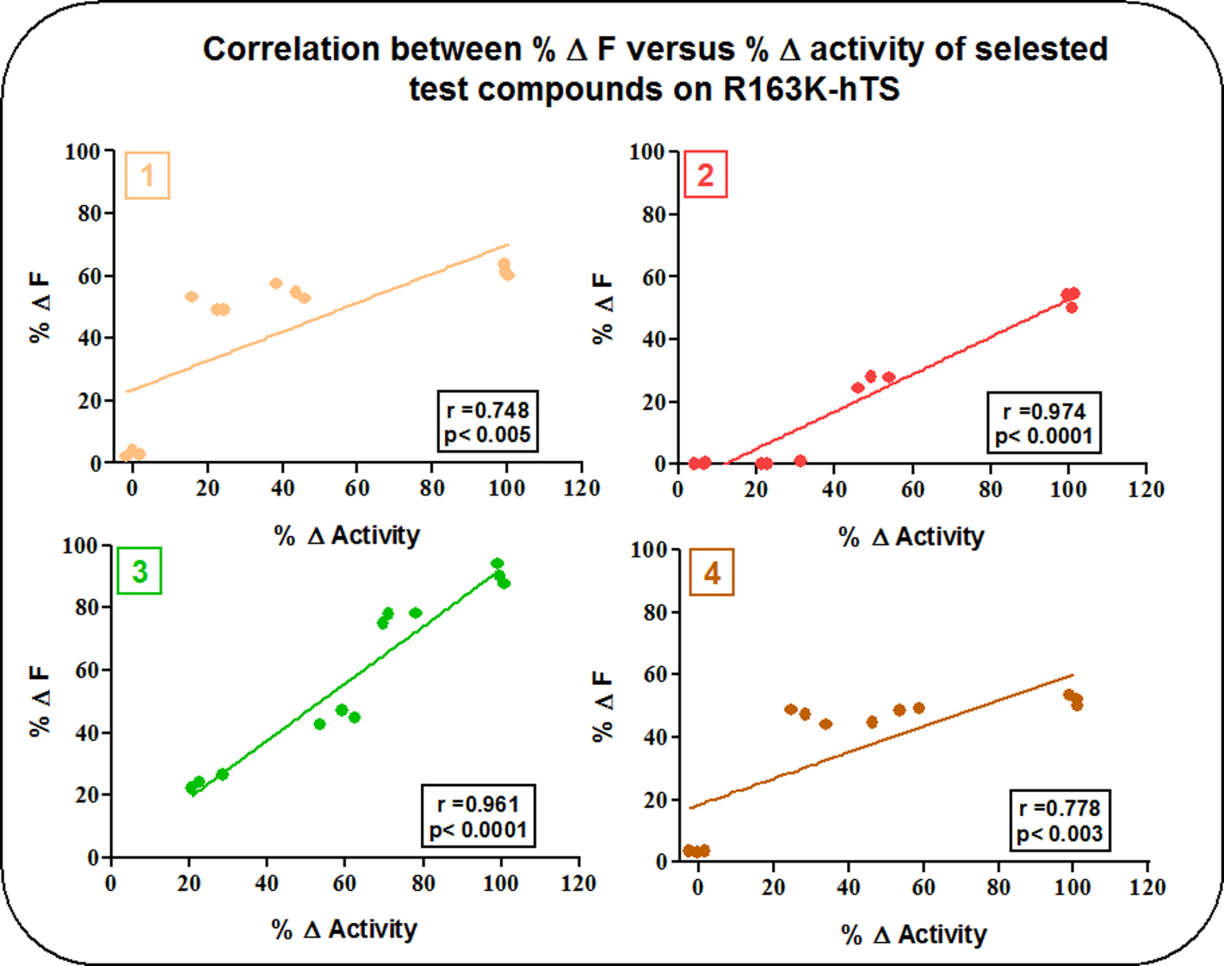
Correlation between activity inhibition and IF studies of compounds (1), (2), (3) and (4) on R163K-hTS.

Fig 5 depicts the correlation between activity inhibition studies and IF studies of compounds (1), (2), (3) and (4) on the active stabilized mutant R163K-hTS. The strong correlation between activity inhibition and Δ IF was retained with compounds (2) and (3) with r values of 0.949 and 0.924, respectively, and p-values < 0.0001. Meanwhile, r values of compounds (1) and (4) were 0.748 and 0.778, respectively, with a p-values < 0.005 and < 0.003, respectively, indicating a weaker correlation.

## Discussion

The current investigation identifies six novel compounds capable of significantly reducing the activity of hTS. These compounds include glutaric acid (2), N-(4-carboxyphenyl) succinamic acid (3), diglycolic anhydride (4), (R)-4-((1-cyclopropylethyl) (phenyl)amino)-4-oxobutanoic acid, (E)-4-((2-(methoxycarbonyl)-4-methythiophen-3-yl)amino)-4-oxobut-2-enoic acid, and 3,4-dihydro-2H-benzo [b][1,4]dioxepine-7-carboxylic acid, in addition to the previously reported diphosphonate PDPA referred to, in the current study, by compound (1) [13, 21]. Previous studies on PDPA reported that its binding to hTS was able to cause significant inhibition of its activity, and stabilize loop 181–197 in an inactive conformation [22, 23]. In the present investigation, PDPA exhibited higher selectivity for inhibition of native hTS relative to active-stabilized R163K-hTS. In contrast, RTX exhibited higher selectivity for inhibition of R163K-hTS, relative to native hTS. This observation is consistent with an interpretation that PDPA is binding to hTS in a distinct mode relative to the active-site inhibitor, RTX. Although the inhibitory potency of PDPA, observed in the current study, is lower than that reported in a previously published work by *Lovelace* and coworkers (2007) [13], it is similar to that reported by Luo et al. [10]. A radioactive assay of catalysis was utilized in the current investigation, to improve sensitivity, so that the basis for differences in the observed potency are unclear. A second discrepancy between the present study and that of Lovelace et al. [13] is the observation that PDPA binding is associated with a downward shift in IF of hTS. In a previous investigation, PDPA binding was associated with an upward shift in IF; however, the concentrations of PDPA employed in IF studies were 1–2 orders of magnitude lower than those required to inhibit enzyme activity [13]. In the present study, the concentrations of PDPA and other ligands, utilized in both IF studies and those of inhibitory potencies, were similar. Compounds (2) and (4) exhibited similar profiles to PDPA in hTS selectivity and induced fluorescence changes upon enzyme binding. While the inhibitory selectivity of RTX differed from the other compounds, it also induced a downward shift in IF. The effect of ligand binding on IF is attributed to the movement of loop 181–197, altering the environment of Trp 182. The data indicate that PDPA and chemotypes are inducing loop movement similar to RTX by binding to conformer(s) unique to hTS, or that loop movement is not the sole determinant of ligand-mediated IF. Interestingly, a Trp residue (Trp 109) occurs in insert 1 in hTS. The region containing insert 1 is disordered in structures of native hTS in inactive conformations and ordered in structures of hTS ternary complexes with RTX, R163K-hTS, and in structures of native hTS in an active conformation [7, 9, 11, 24]. In the structure of M190K-hTS, a mutant strongly stabilized in an inactive conformation, the region containing insert 1 assumes a distinct conformation, relative to hTS in active conformations [8]. Ligand binding to hTS may alter the environment of not only Trp 182, but also Trp 109 and effects on enzyme fluorescence may be ligand-specific and/or concentration-dependent. Collectively, these data suggest that the compounds examined in the present investigation are distinct from RTX, yet it is premature to conclude that they are allosteric inhibitors. As PDPA is reported to exhibit mixed kinetic behavior that is substrate-dependent [13], future studies of inhibition kinetics are warranted.

The higher potency of compounds (1), (3) and (4), reported hereby, compared to compound (2) towards hTS could be rationalized, in part, due to the pK_a_s of the four test compounds. Compound (2) has the highest pK_a_ among the four, while (3) and the acid derivative of (4) have lower pK_a_s. Interestingly, PDPA is the most acidic of the four compounds, but is not significantly more potent as an inhibitor. The data are consistent, as expected, with anionic interactions being an essential determinant of inhibitory potency. Obviously, this is not the only determinant, as the majority of isosteres of (2) exhibited minimal inhibitory activity. An alkyl structure with minimal functionality distinguished (1), (2), and the acid derivative of (4) from the other compounds examined; however, the structural basis for (3) is difficult to evaluate. Anionic interactions are important in enzyme binding, given the predicted binding site of PDPA in crystal structures. PDPA forms hydrogen bonds with Arg215 and Arg50 and these residues were defined as interaction sites. In addition, the region surrounding the PDPA binding site includes Asp49, Arg50, Thr51, Asn183, Arg185, His196, Arg215, Ser216, Tyr258, and Arg175’ (from other subunit). Compound (3) was slightly less potent than PDPA probably due weaker binding with hTS which could be attributed to the absence of phosphate atoms. Phosphate atoms have relatively larger size that is expected to allow better fitting of PDPA to the enzyme, relative to the other compounds. Moreover, the presence of phosphonic acid in PDPA allows the formation of a larger number of H-bonds and probably greater stability of the inactive conformer of hTS, as compared to the carboxylic acid moieties of the other compounds. Recently, and in accord with our results, *Deschamps et al*. were able to demonstrate that charge effects are able to stabilize the substrate-binding pocket of hTS through anionic interactions with Arg residues of both subunits of the dimer enzyme [24]. Hydrolysis of the anhydride will provide ring opening of compound (4) to the acid and is expected to occur in solution, thus acquiring a similar structure to compound (2) with the exception of having an oxygen atom at position 3 rather than a carbon atom. This oxygen atom is expected to form an additional H bond possibly with Arg50 of hTS, allowing for a greater stability of the inactive conformer of hTS. Compounds (1), (2) and (4) have demonstrated lower inhibitory potencies towards R163K-hTS, compared to the wild type of the enzyme, an observation that was absent with compound (3). This finding leads to the hypothesis that this compound might exhibit a different binding mode, or alternatively might have more than one binding site in hTS. This compound, with its relatively large molecular weight and aromatic ring, could possibly expand to the active site, or alternatively to the dimer interface preventing proper dimer assembly. To the best of our knowledge, this is the first experiment to test novel inhibitors of hTS enzyme that have higher activity against TS capable of undergoing conformational switching relative to an active stabilized TS.

Correlation studies of the effects of inhibitors on IF revealed a deviation in behavior of compound (2) towards hTS relative to compounds (1) and (4). This unusual behavior could be attributed to its lower potency towards hTS, as compared to the other two aliphatic compounds. This could in turn suggest a lower binding affinity or a different binding mode, e.g., mixed orthosterism-allosterism. As for the behavior of compound (3) on IF, it could be explained in terms of structural differences attributed to the presence of an aromatic ring not found in the other aliphatic inhibitors that might exhibit a different mode of binding to hTS, as compared to compounds (1) and (4). Such an assumption requires further studies to unravel the exact mechanism by which compound (3) binds to the enzyme. Consistent with the data obtained in studies of inhibition of catalysis, conformational selectivity was also evident in IF studies. At lower concentrations, the inhibitors studied caused a greater decrease in IF of hTS, relative to R163K-hTS. At higher concentrations, this differential effect was lost. At the concentrations of inhibitors that produced maximum inhibition of native hTS `enzyme (90–100%), the ΔIF was approximately 60% with the novel inhibitors compared to about 40% with the active site inhibitor RTX. Thus, the fluorescence response of the native enzyme was more pronounced with the novel inhibitors relative to RTX.

In conclusion, and despite the relatively low potency reported hereby for PDPA and the carboxylate isosteres examined, they represent promising lead agents for selective conformational binding to hTS. Future studies should be directed at determining the structure of co-crystals of inhibitor-bound hTS to obtain evidence for the mode of binding of these inhibitors, as well as models for the design of more potent inhibitors.

## Conclusion

The results of the current work suggest that, despite the relatively low potency reported hereby for PDPA and its isosteres as inhibitors of hTS, they represent promising leads for selectively targeting a conformer(s) in hTS that is distinct from the active conformation stabilized by current hTS inhibitors. Conformer-selective targeting provides an novel approach for overcoming the potential clinical drug resistance associated with active state inhibitors.

## Supporting information

S1 Fig Polyacrylamide gel electrophoresis of native hTS and R163K-hTS after purification. 1 μg denatured enzyme was loaded against a protein ladder with molecular weights 20–100 kDa. The gel was run at 110 volts for 80 minutes and proteins were visualized using 0.25% w/v Coomassie brilliant blue staining.
(TIF)
