# Supplementary figures and images for: Biomolecular study of human thymidylate synthase conformer-selective inhibitors: New chemotherapeutic approach
Source: PLoS One. 2018 Mar 14;13(3):e0193810. doi: 10.1371/journal.pone.0193810 (PMC5851609; doi:10.1371/journal.pone.0193810)

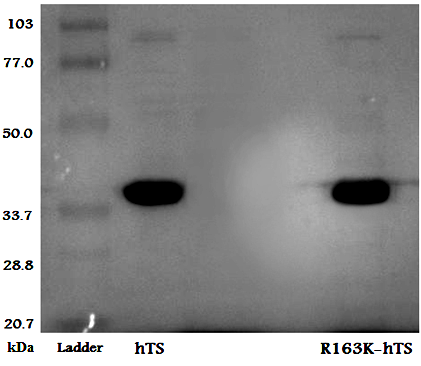

Supplement: S1 Fig — 1 μg denatured enzyme was loaded against a protein ladder with molecular weights 20–100 kDa. The gel was run at 110 volts for 80 minutes and proteins were visualized using 0.25% w/v Coomassie brilliant blue staining. (TIF) [file pone.0193810.s001.tif]
